# Circular RNA circBACH2 plays a role in papillary thyroid carcinoma by sponging miR-139-5p and regulating LMO4 expression

**DOI:** 10.1038/s41419-019-1439-y

**Published:** 2019-02-22

**Authors:** Xiaoyan Cai, Zheng Zhao, Jiangnan Dong, Qiang Lv, Bei Yun, Jiangqi Liu, Yan Shen, Jie Kang, Jun Li

**Affiliations:** 1 Department of General Surgery, Shanghai Gongli Hospital, The Second Military Medical University, Shanghai, China; 2 Department of Ultrasound, Shanghai Gongli Hospital, The Second Military Medical University, Shanghai, China; 3 Department of General Surgery, Sixth People’s Hospital Affiliated to Shanghai Jiao Tong University, Shanghai, China

## Abstract

Circular RNAs (circRNAs) are a class of non-coding RNAs that are broadly expressed in various biological cells and function in regulating gene expression. They are structurally stable and tissue-specific. However, the function of human circRNAs and the role of circRNAs in papillary thyroid carcinoma (PTC) remain to be determined. Herein, the function of circRNA circBACH2 was investigated in human PTC cells. First, we detected the expression of circBACH2 in PTC tissues and PTC cell lines by RT-PCR. FISH was used to confirm the subcellular localization of circBACH2. A luciferase reporter assay and AGO2-RIP was used to confirm the relationship between circBACH2 and miR-139-5p. PTC cells were stably transfected with siRNA against circBACH2 and cell proliferation, migration and invasion were detected to evaluate the effect of circBACH2 in PTC, while tumorigenesis was assayed in nude mice. We found that circBACH2 was highly expressed in PTC tissues and PTC cell lines. Mechanistically, we confirmed that circBACH2 could directly bind to miR-139-5p and relieve suppression of the target LMO4. Functionally, we found that inhibiting circBACH2 expression decreased cell proliferation, migration, and invasion. Finally, down-regulating circBACH2 suppressed the growth of PTC xenografts in nude mice. Our findings indicate that circBACH2 acts as a novel oncogenic RNA that sponges miR-139-5p and can be used as a tumor biomarker of PTC. What’s more, these results revealed that the circBACH2/miR-139-5p/LMO4 axis could be targeted as a potential treatment strategy for PTC.

## Introduction

Thyroid cancer is one of the fastest growing malignant tumors in the world, as its global incidence has tripled over the past 30 years^1^. According to data from the American Cancer Society^2^, ~52,990 new cases of thyroid cancer will occur in 2018, including about 40,900 cases in women; thyroid cancer is responsible for the fifth highest incidence of malignant tumors among women in the United States. According to the “Epidemiological Surveillance and Final Results” data map of the National Cancer Agency of the United States^3^, new cases of thyroid cancer continue to increase at an average annual rate of 5% worldwide. In China, the latest data from the National Cancer Center showed that the annual number of cases of male thyroid cancer is ~22,000, and the number of cases of female thyroid cancer is ~67,900^4^. The high incidence and high rate of growth of thyroid cancer are largely due to the rapid increase in the incidence of papillary thyroid cancer (PTC)^5^. Although PTC generally has a good prognosis, some patients present with early stage extradural invasion, lymph node metastasis, and even distant metastasis and other high-risk conditions, which seriously affect the quality of life of patients. Previous reports suggest that lymph node metastasis may occur in more than 30% of patients in PTC^6^ and distant metastases may occur in 2.6–3.7% of patients^7^. The occurrence of PTC invasion and metastasis indicates a poor prognosis, and often the condition cannot be effectively controlled, commonly leading to death. Therefore, an in-depth study of the mechanism of PTC invasion and metastasis has important theoretical significance and potentially valuable clinical implications.

Circular RNA (circRNA) is a class of non-coding RNA that is broadly found in mammals. It is mainly involved in gene regulation in vivo^8–10^. Most circRNAs are derived from the gene’s exon region, but also a small portion are formed by intron cleavage^11,12^. CircRNAs are widely involved in human physiological and pathological processes and can be used in various ways, such as: (1) microRNA (miRNA) sponges; (2) to interact with protein binding; (3) in a range of pathways including translation into peptides. Several circRNAs have been found to contain at least one miRNA binding site. Therefore, they can be used as RNA “sponges” to adsorb miRNAs, thereby regulating the expression of downstream target genes that are inhibited by miRNAs through the mechanism of competing endogenous RNAs^13^. In tumor research, the use of circRNAs as miRNA “sponges” to regulate downstream target genes has been widely reported^14–16^. Previous research suggests that circBACH2 (hsa_circ_0001627) shows aberrant expression, but it remains to be determined whether circBACH2 plays a role in the progression of PTC^17^. Over the last decade, an increasing volume of research has focused on the regulatory properties of miRNA and their contribution to tumorigenesis and metastasis^16,18^. However, the regulatory roles of circBACH2 and its potential role as an “miRNA sponge” in PTC are still largely unclear.

In our research, we analyzed the expression of circBACH2 in PTC tissues and found that circBACH2 is significantly upregulated in PTC and closely linked with the survival of PTC patients. We found that circBACH2 may act as a sponge of miR-139-5p to upregulate the level of LMO4 and therefore promote PTC development. Hence, our results might provide new evidence for the development of clinical therapeutic strategies against PTC.

## Results

### circBACH2 is highly expressed in PTC tissues and PTC cell lines

**Fig. 1:**
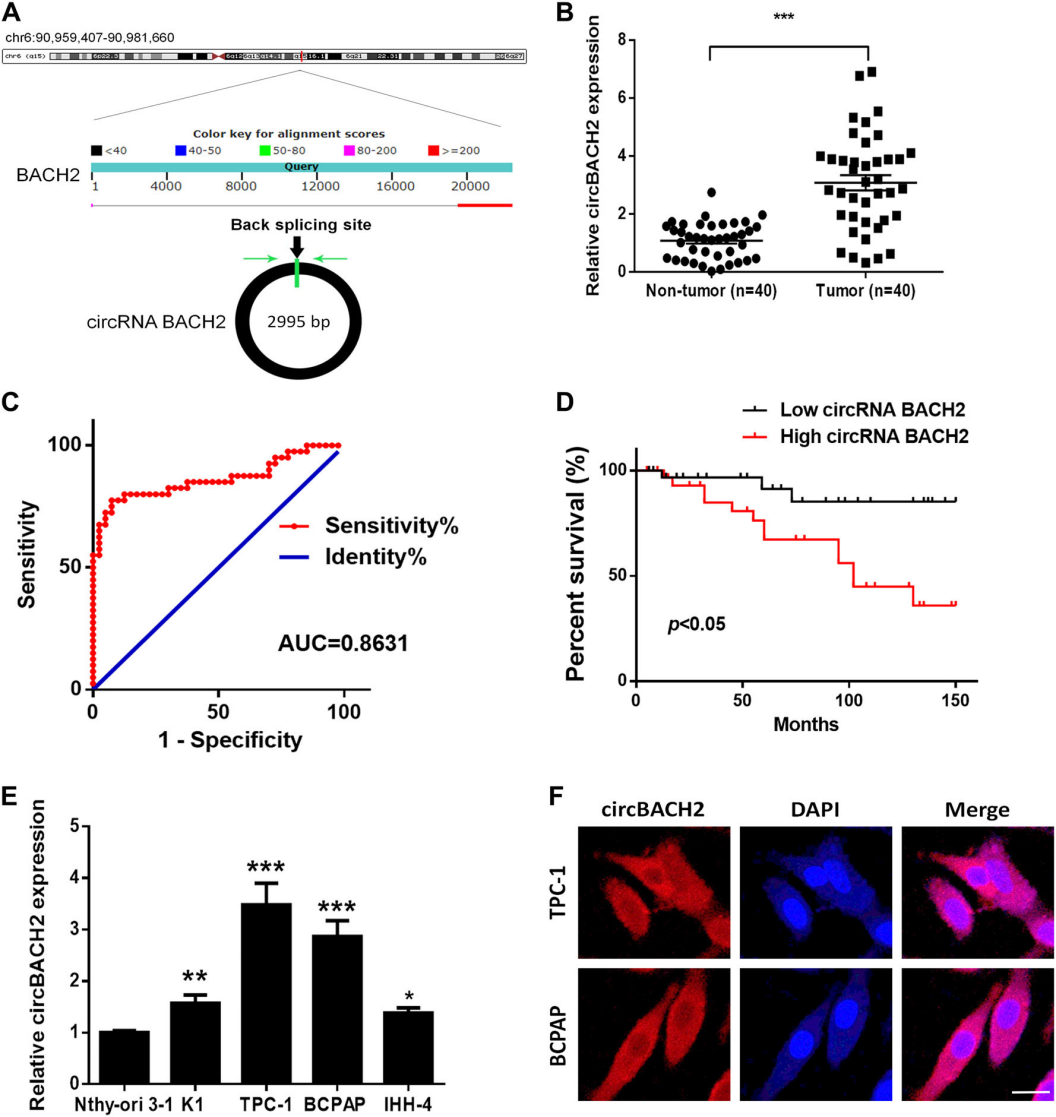
CircBACH2 is highly expressed in PTC tissues and PTC cell lines. **a** The genomic loci of the *BACH2* gene and circBACH2. Green arrow indicates back-splicing. **b** CircBACH2 levels were detected by RT-PCR in PTC tissues (*n* = 40) compared with paratumor tissue samples (*n* = 40). **c** The area under the ROC curve was 0.8631 (95% CI = 0.7774–0.9489, *P* < 0.0001). **d** Kaplan–Meier survival curve of patients with low and high expression of circBACH2. **e** CircBACH2 expression levels in PTC cell lines and human thyroid follicular epithelial cells (*n* = 3) were analyzed by RT-PCR. **f** Fluorescence in situ hybridization assay was conducted to determine the subcellular localization of circBACH2. Scale bar, 20 μm. Data indicate the mean ± SD, *n* = 3. *P < 0.05, **P < 0.01, ***P < 0.001 vs. control

A previously reported high-throughput microarray assay revealed that the circRNA_100395/miR-141-3p/miR-200a-3p axis may be related to the etiopathogenesis of PTC^17^. The study also demonstrated the high expression of circBACH2 in PTC. CircBACH2 (hsa_circ_0001627) is derived from exon 2 of the BACH2 gene, whose spliced mature sequence length is 2995 bp. This gene is located on chromosome 6: 90959407–90981660 (Fig. 1a). We examined the expression level of circBACH2 in PTC tissues and cell lines. We found that circBACH2 is highly expressed in 40 PTC tissues compared with matched paratumor tissues (Fig. 1b). We used the ROC curve to examine the diagnostic value of circBACH2 in PTC tissues compared with paratumor tissues, and found the area under the ROC curve (AUC) to be 0.8631 (95% CI = 0.7774–0.9489, *P* < 0.0001; Fig. 1c). Then, the correlations of circBACH2 expression and special clinicopathological parameters and prognosis of PTC were analyzed, as shown in Table 1. Furthermore, PTC patients with low expression of circBACH2 displayed obviously longer overall survival times than those with high expression of circBACH2 according to Kaplan–Meier survival curve analysis (*P* < 0.05) (Fig. 1d). Similarly, in PTC cell lines (K1, IHH-4, BCPAP, and TCP1), circBACH2 expression was higher compared with human thyroid follicular epithelial cells Nthy-ori 3–1 (Fig. 1e). A fluorescence in situ hybridization (FISH) assay revealed that circBACH2 predominately localized in the cytoplasm (Fig. 1f). These results showed that the high expression of circBACH2 is an early event in PTC development and plays an important role in PTC progression.

**Table 1:** Relationship between circBACH2 expression and the clinical pathological characteristics of PTC patients (*n* = 40)

Clinic pathological features		No. of cases	circBACH2 (*n*, %)		*p-*value
			Low	High	
Gender	Male	24	12	12	>0.05
	Female	16	9	7	
Age	≤45	29	13	16	>0.05
	>45	11	5	6	
Extra thyroidal extension	Negative	12	4	8	>0.05
	Positive	28	11	17	
Tumor size	≤1	31	22	9	<0.05
	>1	9	2	7	
TNM stage	I/II	23	17	6	<0.05
	III/IV	17	5	12	
Lymph node metastasis	≤45	18	13	5	<0.05
	>45	22	7	15	
Nodular Goiter	Negative	26	12	14	>0.05
	Positive	14	6	8	

### circBACH2 can function as a miRNA sponge to negatively control miR-139-5p in PTC cell lines

**Fig. 2:**
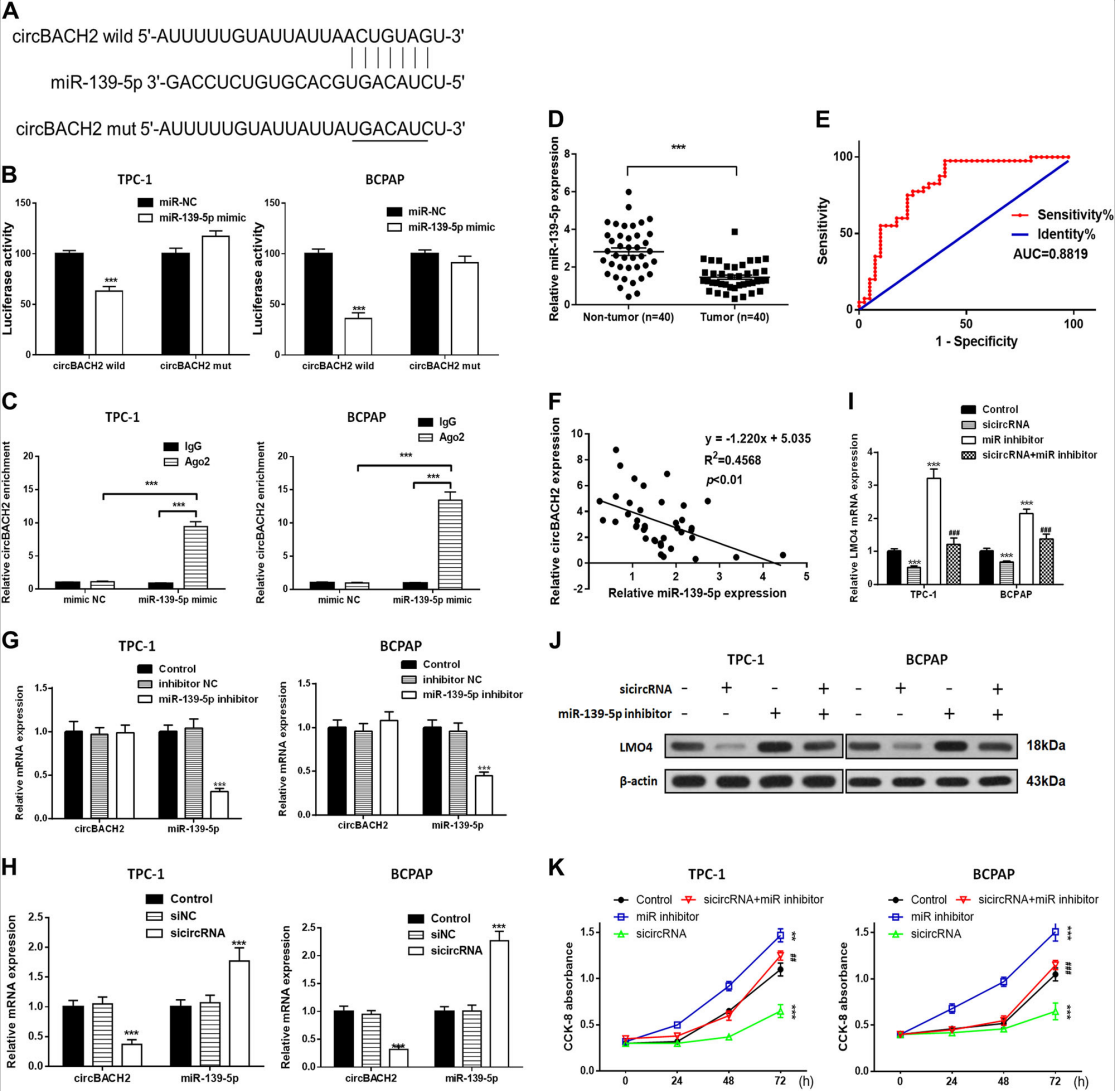
Silencing of circBACH2 inhibits cell proliferation in TPC-1 and BCPAP cells. **a** Wild-type (wt) and mutated (mut) circBACH2 was transfected into TPC-1 and BCPAP cells with or without synthetic miR-139-5p mimics. **b** Relative luciferase activity was detected by luciferase assays in TPC-1 and BCPAP cells. **c** Anti-AGO2 RIP was performed in TPC-1 and BCPAP cells transfected with miR-139-5p mimics or miR-NC, followed by RT-PCR to detect circBACH2. **d** miR-139-5p levels were detected by RT-PCR in PTC tissues (*n* = 40) compared with paratumor tissue samples (*n* = 40). **e** The area under the ROC curve was 0.8819 (95% CI = 0.8254–0.9384, *P* < 0.0001). **f** The correlation between miR-139-5p and circBACH2 expression in PTC tissues was determined by Spearman’s correlation analysis. **g–i** The expression of miR-139-5p and circBACH2 was determined by RT-PCR in TPC-1 and BCPAP cells, in which sicircRNA, miR-139-5p inhibitor, or both, were suppressed. **j** The expression of LMO4 was determined by RT-PCR and western blotting in TPC-1 and BCPAP cells in which sicircRNA, miR-139-5p inhibitor, or both, were suppressed. **k** Cell viability was determined by a CCK-8 assay in TPC-1 and BCPAP cells. Data indicate the mean±SD, *n* = 3. *P < 0.05, **P < 0.01, ***P < 0.001 vs. control and ^##^*P* < 0.01, ^###^*P* < 0.001 vs. sicircRNA

To investigate the relationship between miR-139-5p and circBACH2, we searched for putative miR-139-5p binding sites in circBACH2 (Fig. 2a) and generated luciferase reporter constructs in which these putative binding sites were mutated. Mutant (mut) and wild-type (wt) luciferase reporter constructs were transfected into TPC-1 and BCPAP cells together with miR-139-5p mimics or controls (miR-NC). miR-139-5p mimics significantly inhibited luciferase activity in cells transfected with wild-type constructs, but luciferase activity was not affected in cells transfected with mutant constructs (Fig. 2b), indicating that miR-139-5p directly targets circBACH2. It is well-known that mRNA translation is suppressed by miRNAs in an AGO2-dependent manner by binding of miRNAs to target sequences. We performed anti-AGO2 immunoprecipitation (RIP) in TPC-1 and BCPAP cells and overexpressed miR-139-5p to pull down circBACH2 by means of anti-AGO2 antibodies or control IgG, and used RT-PCR to analyze circBACH2 levels. The circBACH2 pulled down with anti-Ago2 was enriched in miR-139-5p-overexpressing cells compared with miR-NC (Fig. 2c), which revealed that circBACH2 could play a role as a miR-139-5p sponge.

### Silencing of circBACH2 inhibits PTC cell growth, migration, and invasion by promoting miR-139-5p in vitro

**Fig. 3:**
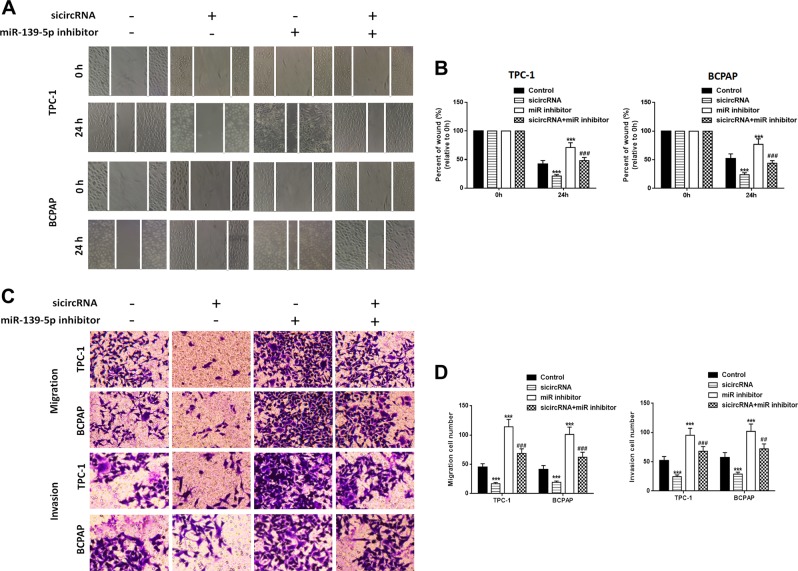
Silencing of circBACH2 inhibits cell migration and invasion in TPC-1 and BCPAP cells. **a**, **b** Wound healing assay was performed in TPC-1 and BCPAP cells in which sicircRNA, miR-139-5p inhibitor, or both, were suppressed and was quantified. **c**, **d** Cell migration and invasion were assessed by transwell assays and quantified. Data indicate the mean ± SD, *n* = 3. **P < 0.01, ***P < 0.001 vs. control and ^##^*P* < 0.01, ^###^*P* < 0.001 vs. sicircRNA

We detected the level of miR-139-5p in PTC patients’ tissues. In contrast to circBACH2, miR-139-5p expression was higher in paratumor tissues compared with PTC tissues. We used the ROC curve to examine the diagnostic value of miR-139-5p in PTC tissues compared with paratumor tissues (Fig. 2d) and found the AUC to be 0.8819 (95% CI = 0.8254–0.9384, *P* < 0.0001) (Fig. 2e). In addition, Spearman rank correlation assessment indicated an inverse correlation between circBACH2 and miR-139-5p in PTC tissues (Fig. 2f). siRNA against circBACH2 (sicircRNA) was constructed and RT-PCR revealed that the level of circBACH2 in PTC cells was downregulated and miR-139-5p was upregulated compared with the negative control (NC) or control groups after transfection with sicircRNA for 48 h (Fig. 2g). Then, we treated PTC cells with an miR-139-5p-specific inhibitor, and the RT-PCR results showed that miR-139-5p was downregulated compared with the NC or control groups, while it had no effect on circBACH2 expression. Next, we detected the LMO4 level in PTC cells in which sicircRNA, the miR-139-5p inhibitor, or both, were suppressed. The RT-PCR and western blot results showed that silencing circBACH2 downregulated LMO4 expression and miR-139-5p inhibitor upregulated LMO4 expression (Fig. 2i, j). Cell viability was measured using a CCK-8 assay in PTC cells in which circBACH2, miR-139-5p, or both, were suppressed. The results showed that inhibition of miR-139-5p reversed the decrease in cell proliferation induced by circBACH2 in the PTC cell lines (Fig. 2k). A wound healing assay indicated that circBACH2 silencing led to quicker closing of scratch wounds compared with the control group, while inhibition of miR-139-5p reversed these results (Fig. 3a, b). The migration and invasion abilities of PTC cells were investigated using transwell assays. We found that circBACH2 silencing significantly decreased the migration and invasion in PTC cells compared with control cells, and these effects were reversed by inhibition of miR-139-5p (Fig. 3c, d).

### Ectopic expression of miR-139-5p inhibits PTC cell growth, migration and invasion by inhibiting LMO4 in vitro

**Fig. 4:**
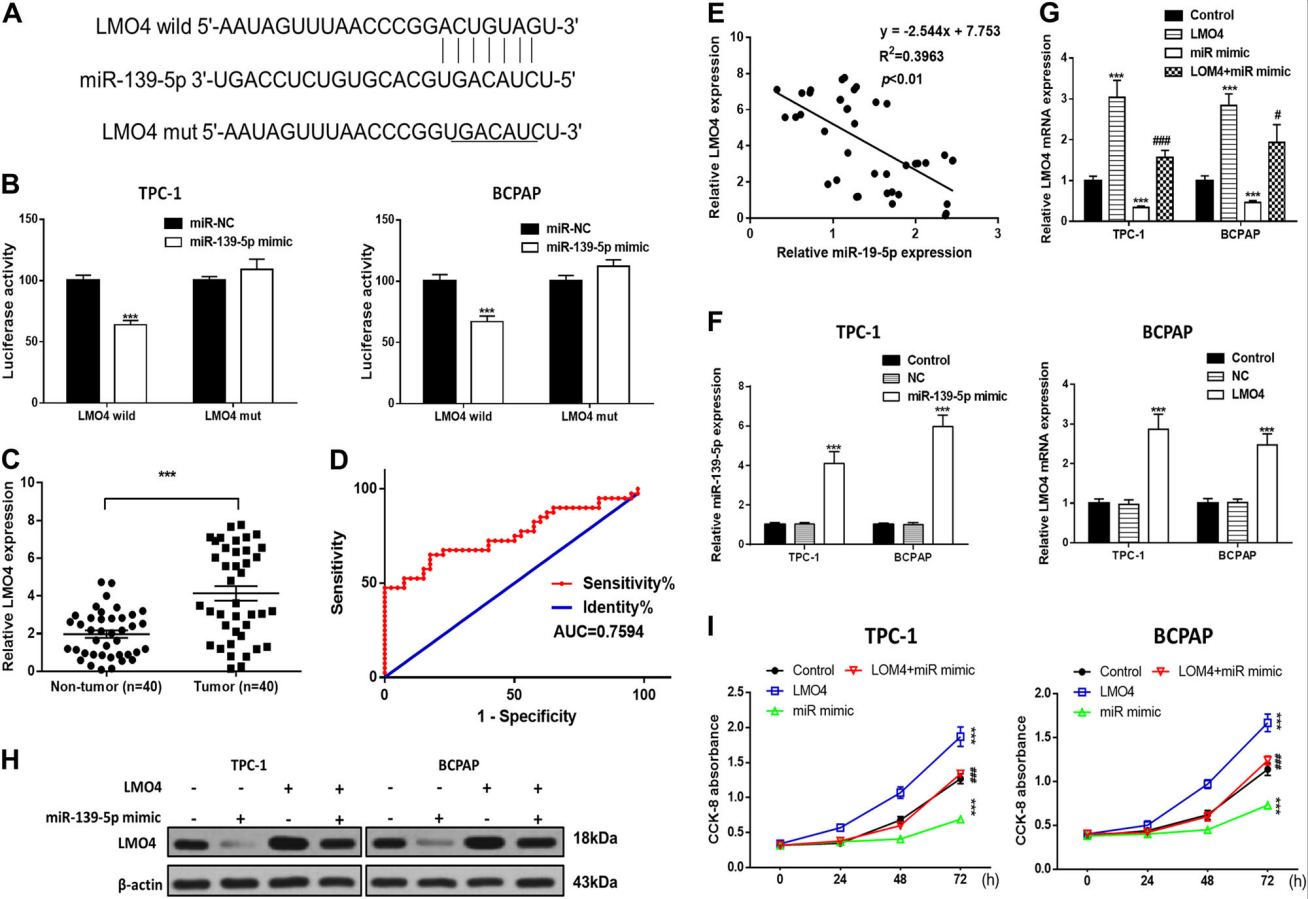
miR-139-5p involvement in PTC cells is mediated by the modulation of LMO4. **a** The binding sites of miR-139-5p in the 3′UTR of LMO4 were predicted. The mutated version of the LMO4 3′UTR is also shown. **b** The relative luciferase activity was determined 48 h after transfection with the miR-139-5p mimic/NC or the 3′UTR of LMO4 wt/mut in TPC-1 and BCPAP cells. **c** LMO4 levels were detected by RT-PCR in PTC tissues (*n* = 40) compared with paratumor tissue samples (*n* = 40). **d** The area under the ROC curve was 0.7594 (95% CI = 0.6519–0.8669, *P* < 0.0001). **e** The correlation between miR-139-5p and LMO4 expression in PTC tissues was determined by Spearman’s correlation analysis. **f** The expression of miR-139-5p and LMO4 was determined by RT-PCR in TPC-1 and BCPAP cells, in which miR-139-5p or LMO4 were overexpressed. **g**, **h** The expression of LMO4 was determined by RT-PCR and western blotting in TPC-1 and BCPAP cells in which miR-139-5p, LMO4, or both, were overexpressed. **i** Cell viability was determined by a CCK-8 assay in TPC-1 and BCPAP cells. Data indicate the mean ± SD, *n* = 3. *P < 0.05, **P < 0.01, ***P < 0.001 vs. control and ^#^*P* < 0.05, ^###^*P* < 0.001 vs. miR-139-5p mimic

**Fig. 5:**
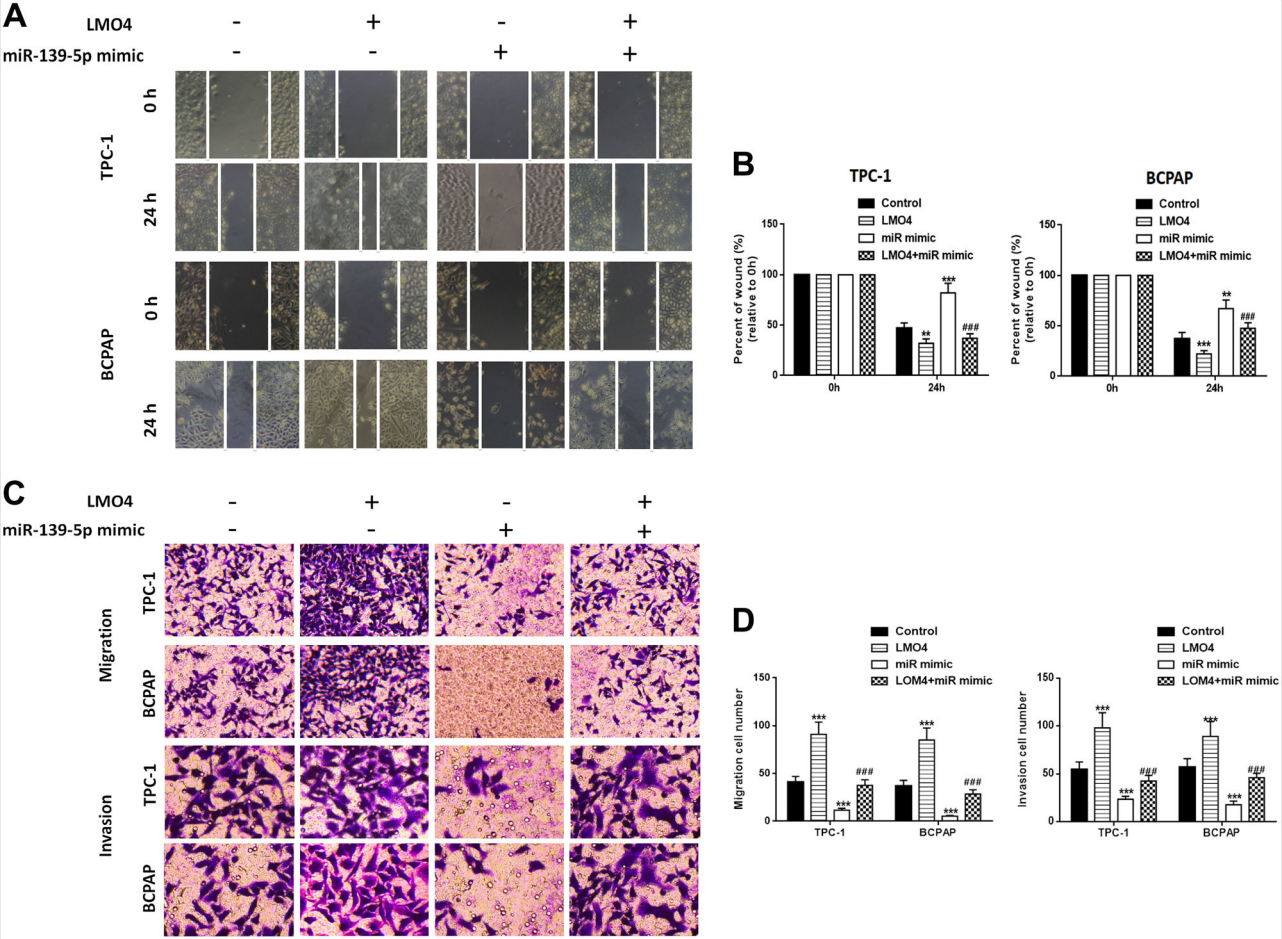
miR-139-5p inhibits cell migration and invasion in TPC-1 and BCPAP cells, which is reversed by LMO4. **a**, **b** Wound healing assay was performed in TPC-1 and BCPAP cells and quantified. **c**, **d** Cell migration and invasion were assessed by Transwell assays and quantified. Data indicate the mean ± SD, *n* = 3. **P < 0.01, ***P < 0.001 vs. control and ^###^*P* < 0.001 vs. miR-139-5p mimic

To investigate the relationship between miR-139-5p and LMO4, we searched for putative miR-139-5p binding sites in the 3ʹUTR of LMO4 (Fig. 4a) and generated luciferase reporter constructs in which these putative binding sites were mutated. Mutant (mut) and wild-type (wild) luciferase reporter constructs were transfected into TPC-1 and BCPAP cells together with miR-139-5p mimics or controls (miR-NC). miR-139-5p mimics significantly inhibited luciferase activity in cells transfected with wild-type constructs, but luciferase activity was not affected in cells transfected with mutant constructs (Fig. 4b), indicating that miR-139-5p directly targets LMO4. Next, we detected the level of LMO4 in PTC patients’ tissues. The results showed that LMO4 expression was lower in paratumor tissues compared with PTC tissues. We used the ROC curve to examine the diagnostic value of LMO4 in PTC tissues compared with paratumor tissues (Fig. 4c) and found the AUC to be 0.7594 (95% CI = 0.6519–0.8669, *P* < 0.0001; Fig. 4d). Furthermore, Spearman rank correlation assessment indicated an inverse correlation between LMO4 and miR-139-5p, confirming that miR-139-5p downregulates LMO4 in PTC tissues (Fig. 4e). To examine the role of miR-139-5p and LOM4 in PTC cells, miR-139-5p and LMO4 were overexpressed. The RT-PCR results showed that miR-139-5p was upregulated compared with the NC or control groups after transfection with the miR-139-5p mimic, while LMO4 was also upregulated after transfection with the overexpression vector (Fig. 4f). Next, we detected LMO4 levels in PTC cells in which LMO4, the miR-139-5p mimic, or both, were overexpressed. The RT-PCR and western blot results showed that miR-139-5p mimic downregulated LMO4 expression and LMO4 overexpression upregulated LMO4 expression (Fig. 4g, h). Cell viability was measured using the CCK-8 assay in PTC cells in which LMO4, the miR-139-5p mimic, or both, were overexpressed. The results showed that overexpression of LMO4 reversed the decrease in cell proliferation induced by the miR-139-5p mimic in the PTC cell lines (Fig. 4i). A wound healing assay indicated that the miR-139-5p mimic led to slower closing of scratch wounds compared with the control group, while overexpression of LMO4 reversed these results (Fig. 5a, b). The migratory and invasive abilities of PTC cells were investigated using transwell assays. miR-139-5p mimic significantly decreased migration and invasion in PTC cells compared with control cells, and these effects were reversed by overexpression of LMO4 (Fig. 5c, d).

### Inhibiting miR-139-5p reverses the effect of silencing of circBACH2 on suppressing tumor growth in vivo

**Fig. 6:**
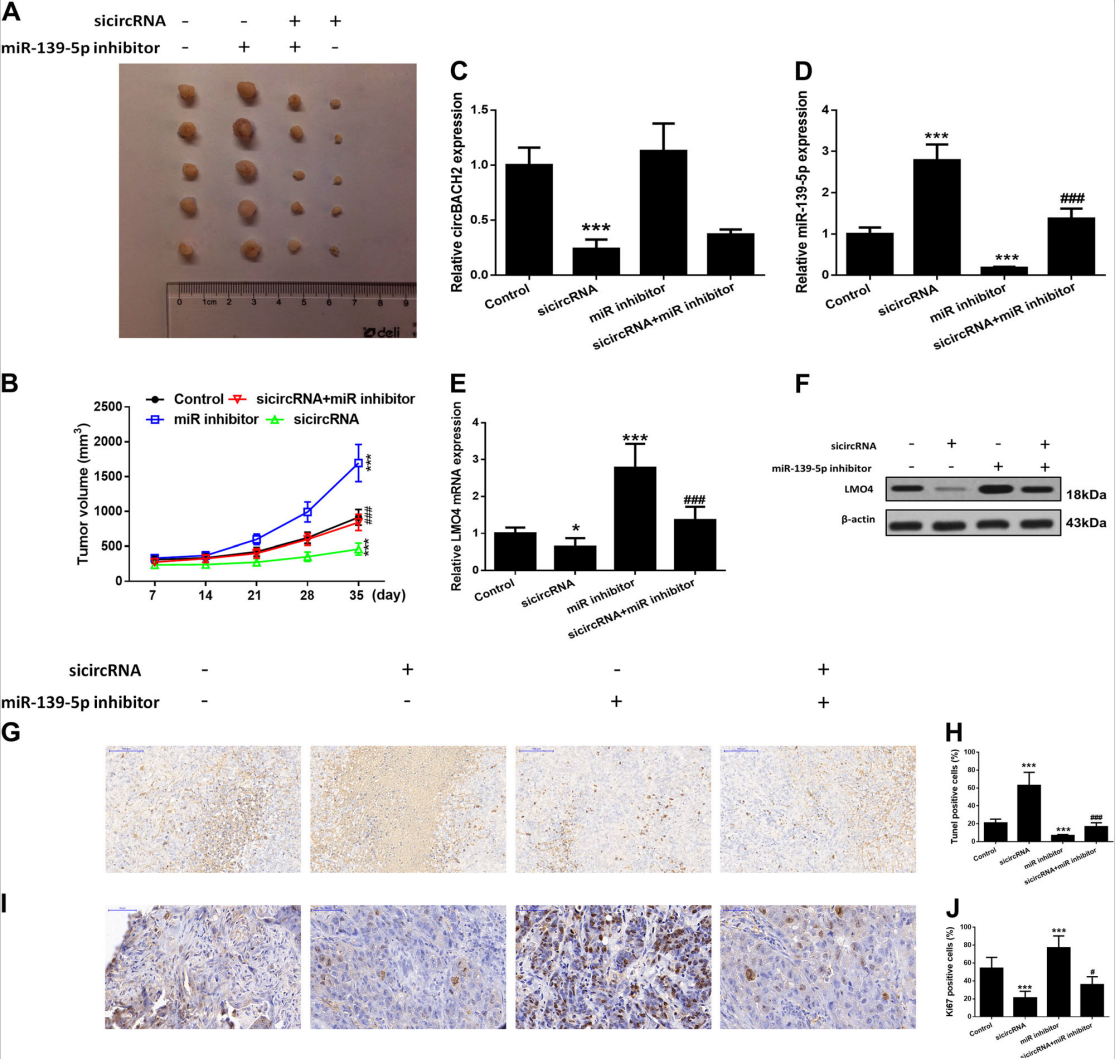
Effects of circBACH2 or miR-139-5p in a tumor xenograft model. TPC-1 cells stably inhibiting circBACH2 or miR-139-5p were inoculated subcutaneously into the right flank regions of 4-week-old male BALB/c nude mice. **a** Representative images of xenograft tumors isolated from nude mice in the different groups. **b** Tumor sizes in the different groups. **c**, **d** The expression of circBACH2 and miR-139-5p was detected by RT-PCR in the indicated groups of tumors. **e**, **f** The expression of LMO4 was detected by RT-PCR and western blotting in the indicated groups of tumors. **g**, **h** The TUNEL assay (400×) was performed to determine the apoptotic indices and quantified. **i**, **j** The Ki-67 assay (400×) was performed by immunohistochemistry and quantified. Scale bar, 100 μm. Data indicate the mean±SD, *n* = 5. **P < 0.01, ***P < 0.001 vs. control and ^###^*P* < 0.001 vs. sicircRNA

The effects of circBACH2 and miR-139-5p on tumor growth in vivo were measured in a tumor xenograft model generated by implanting TCP-1 cells subcutaneously into the left flank of nude mice. The results indicated that silencing of circBACH2 suppressed tumor volumes, whereas concomitant inhibition of miR-139-5p inverted the results for circBACH2, restoring tumor volumes compared to control levels (Fig. 6a, b). RT-PCR showed that silencing of circBACH2 downregulated circBACH2 levels and upregulated miR-139-5p levels, while inhibition of miR-139-5p reversed these results (Fig. 6c, d). Western blot and RT-PCR detection of LMO4 expression in tumors showed that silencing of circBACH2 downregulated LMO4 and partially inhibited the effects of miR-139-5p inhibition (Fig. 6e, f). We also assessed the stages of apoptosis in tumor tissue by a TUNEL assay. Tissue samples from tumors transfected with silencing circBACH2 contained a higher level and inhibition of miR-139-5p and a lower level of apoptotic cells (Fig. 6g, h). Ki67 is used as a marker of cell proliferation as it is absent in resting cells. The number of proliferating cells detected by a Ki67 immunohistochemistry assay was lower in tissue silencing circBACH2 (Fig. 6h, i). These results showed that PTC cells in which circBACH2 was silenced could effectively inhibit tumor development in vivo, while inhibition of miR-139-5p could reverse this result.

## Discussion

To date, only a few circRNAs have been reported. Here, we report a new circRNA, designated circBACH2, the expression of which was increased in human PTC and was related to the survival rate of patients. We used 40 patients to validate the correlation between circBACH2 and overall survival time. In future research, a larger cohort of patients should be used to assess the clinical potential of this candidate biomarker. It has been reported that BRAF V600E can promote PTC tumorigenesis by altering methylation and hence the expression of numerous important genes^19^. The correlation between the BRAF V600E mutational status of the patients and the expression of circBACH2 needs to be validated. The differences between TPC-1 cells and the BRAF V600E mutant cell line BCPAP also requires further investigation. We also discovered that downregulation of circBACH2 could suppress cell proliferation and migration as well as facilitate tumor growth in vitro and in vivo. Mechanistically, circBACH2 could function as a sponge by harboring miR-139-5p and thereby abolishing the suppressive effect on the target gene LMO4 in PTC development. Thus, our data suggest that circBACH2 could play an important role in the pathogenesis and progression of PTC.

The abnormal expression of miR-139-5p has been reported in many types of cancer such as lung cancer, colorectal cancer, prostate cancer, breast cancer, and acute granulocyte lymphoma^20,21^. In colorectal cancer, Li and colleagues^22^ found that the expression of miR-139-5p in tumor tissues was significantly lower than that in paratumor tissues and correlated with tumor stage; furthermore, upregulating miR-139-5p influenced the metastatic potential and drug resistance of colorectal cancer cells through the EMT and BLC-2 apoptotic pathways. Cao and coworkers^23^ found that expression of miR-139-5p in colorectal cancer cells can lead to the downregulation of PDE4D and the upregulation of cAMP, resulting in BIM-mediated cell growth arrest and the inhibition of tumor growth. Catanzaro and colleagues^24^ reported downregulation of miR‐139‐5p in cell proliferation as a result of depression of PI3K/AKT signaling in low‐grade gliomas. We found that miR-139-5p was expressed at a significantly lower level in tumor tissues than in paratumor tissues. Next, we verified that circBACH2 had an endogenous sponge-like effect on miR-139-5p in PTC. First, we found that circBACH2 levels are negatively correlated with miR-139-5p levels in PTC patients’ tissues. Furthermore, bioinformatics prediction and a luciferase reporter assay showed that circBACH2 and the LMO4 3ʹUTR share identical miR-139-5p response elements and might therefore bind competitively to miR-139-5p. Third, circBACH2 could bind directly to miR-139-5p in an AGO2-dependent manner. Finally, circBACH2 could control the LMO4 level by provoking miR-139-5p. It has recently been reported that circRNAs can act as miRNA sponges to negatively control miRNA. Approximately 85% of circRNAs are aligned in the sense orientation to known protein-coding genes, and span 1–5 exons. The majority of circRNAs can function as sponges, via a mechanism of back-splicing, as they are enriched in miRNA binding sites. They can also competitively bind to miRNAs and decrease the activity of miRNAs^25,26^. Our results further showed that circRNAs can serve as competitive endogenous RNAs and play an important role in PTC development.

In recent years, increasing evidence has confirmed the important role of LIM-only protein 4 (LMO4) in tumorigenesis. LMO4 was first discovered by Racevskis and colleagues^27^ as an autoantigenic protein of human breast cancer cells that can cause cell proliferation, enhance cell invasiveness, and directly lead to canceration of breast cells. Yu and coworkers^28^ found that the transcriptional level of LMO4 is higher in invasive pancreatic ductal carcinoma than in normal pancreatic ductal cells and some other types of pancreatic cancer; meanwhile, Murphy and colleagues^29^ also found that the prognosis of pancreatic cancer with low LMO4 expression is poor, indicating that LMO4 plays a complex role in the development of pancreatic cancer. Mizunuma and colleagues^30^ found that the LMO4 and Ldb1 complexes were highly expressed at the invasive front of oral cancer, and the expression level of LMO4 was negatively correlated with the stage of differentiation, suggesting that LMO4 might promote cell differentiation through interaction with Ldb1 and promote tumor progression. Expression of LMO4 in primary prostate cancer is also upregulated, but expression levels decline during the progression of this cancer;^31^ however, the mechanism for this has not yet been reported. In our research, we discovered that miR-139-5p can interact with the 3’UTR of LMO4, then silence LMO4 at the post-transcriptional level. Overexpression LMO4 reversed the overexpression of miR-139-5p and induced cell growth, migration, and invasion inhibition. Taken together, these results revealed the tumor-suppressor role of the miR-139-5p/LMO4 pathway in PTC.

## Conclusion

Our results provide evidence that circBACH2 functions as a novel oncogenic circRNA by sponging miR-139-5p, and indicate that circBACH2 is a promising prognostic biomarker in PTC. Our results also revealed that targeting the circBACH2/miR-139-5p/LMO4 axis is a potential treatment strategy for PTC.

## Materials and methods

### Clinical specimens and cell lines

PTC tumor and normal tissues were obtained from patients who were diagnosed with PTC and who had undergone surgery at Pudong New Area Gongli Hospital between 2014 and 2017. In total, 40 pairs of tissue samples were freshly frozen in liquid nitrogen and stored at −80 °C until RNA extraction. The use of tissues for this study was approved by the Ethics Committee of Pudong New Area Gongli Hospital, Shanghai Second Military Medical University.

Human PTC cell lines K1, IHH-4, BCPAP, and TCP1 and human thyroid follicular epithelial cells Nthy-ori 3-1 were obtained from Shanghai Institute of Cell Biology (Shanghai, China) and were cultured in RPMI-1640 medium (HyClone, Logan, UT, USA) with 10% fetal bovine serum (FBS) and 1% antibiotics (both from Gibco-BRL, Gaithersburg, MD, USA).

### Fluorescence in situ hybridization

Specific probes to the circBACH2 sequence labeled with cy5 were used for in situ hybridization as previously described^32^. Nuclei were counterstained with 4,6-diamidino-2-phenylindole (DAPI). All procedures were conducted according to the manufacturer’s protocol (Genepharma, Shanghai, China).

### CircRNA analysis and target prediction

Prediction of the hsa_circ_0001627-miRNA-target gene was performed using the https://circinteractome.nia.nih.gov/ website.

### Luciferase reporter assay

To construct luciferase reporter vectors, the 3ʹ-UTR of circBACH2 and LMO4 cDNA fragments containing the predicted potential miR-139-5p binding sites were amplified by PCR and sub-cloned downstream of the luciferase gene in the pmirGlo Dual-Luciferase vector (Promega, Fitchburg, WI, USA). The 3ʹ-UTR of circBACH2 containing the binding sites for miR-139-5p was amplified from a cDNA library with the following primers: forward, 5ʹ-CTCGAGATTTTTGTATTATTAACTGTAGT-3ʹ and reverse, 5ʹ-GCGGCCGCTTAGCAGGAAGGCACTATT-3ʹ. The mutant 3ʹ-UTR of circBACH2, in which seven nucleotides were mutated within the binding sites, was amplified using the following primer sequences: forward, 5ʹ-CTCGAGATTTTTGTATTATTATGACATCT-3ʹ and reverse, 5ʹ-GCGGCCGCTTAGCAGGAAGGCACTATT-3ʹ. The 3ʹ-UTR of LMO4 containing binding sites for miR-139-5p was amplified from a cDNA library with the following primers: forward, 5ʹ- CTCGAGAATAGTTTAACCCGGACTGTAGT-3ʹ and reverse, 5ʹ-GCGGCCGCTTTTTTCATTTTCTCTACAGTC-3ʹ. The mutant 3ʹ-UTR of LMO4, in which seven nucleotides were mutated in the binding sites, was amplified using the following primer sequences: forward, 5ʹ-CTCGAGAATAGTTTAACCCGGTGACATCT-3ʹ and reverse, 5ʹ-GCGGCCGCTTTTTTCATTTTCTCTACAGTC-3ʹ.

For luciferase assays, TPC-1 and BCPAP cells were cultured in 24-well plates and co-transfected with 50 ng of the corresponding vectors containing firefly luciferase together with 25 ng of miR-139-5p or the control. Transfection was performed using Lipofectamine 2000 reagent (Invitrogen, Carlsbad, CA, USA). At 48 h post-transfection, relative luciferase activity was calculated by normalizing the Firefly luminescence to the Renilla luminescence using a Dual-Luciferase Reporter Assay (Promega, Fitchburg, WI, USA) according to the manufacturer’s instructions.

### Cell transfection

To assess circBACH2 expression, siRNA against circBACH2 vector was constructed by GenePharma. Then PTC cells were transfected with the circBACH2 downregulation vector at 50 nM by using Lipofectamine 2000 (Invitrogen).

To assess miR-139-5p expression, an miR-139-5p overexpression vector (miR-mimic) and negative control (miR-NC) were created by GenePharma. Then, PTC cells were transfected with either the miR-139-5p overexpression construct or miR-NC at 50 nM by using Lipofectamine 2000 (Invitrogen). Cells were used for miR-139-5p expression analysis or other experiments after 48 h of transfection. For miR-139-5p inhibition, PTC cells were treated with miR-139-5p inhibitor for 48 h before miR-139-5p expression analysis or other experiments.

For LMO4 overexpression, an LMO4 overexpression vector was constructed by GenePharma. Then, PTC cells were transfected with either the LMO4 overexpression vector or the negative control at 50 nM by using Lipofectamine 2000 (Invitrogen). All steps were performed according to the manufacturer’s instructions.

### Quantitative PCR analysis

RNA was isolated from PTC cells using TRIzol reagent (Invitrogen). cDNA was synthesized from 1 μg of total RNA in 21-μl reaction volumes using oligo dT18 primers and SuperScript reverse transcriptase. PCR amplification was carried out with Taq DNA polymerase (TaKaRa, Tokyo, Japan) using 1 μL of the first-strand cDNA as template. The amplification reactions were run with 30 thermocycles of 30 s at 94 °C, 30 s at 55 °C, and 30 s at 72 °C. The expression levels were calculated by the 2^˗ΔΔCT^ method^33^.

### Protein isolation and western blot analysis

Proteins (50 μg) from lysed cells were separated by 10% SDS-PAGE and transferred to nitrocellulose membranes, then blocked for 2 h. Next, the membranes were incubated overnight with primary antibodies, followed by horseradish peroxidase (HRP)-conjugated secondary antibodies. The protein bands were visualized using ECL Plus Detection Reagent (Applygen, Beijing, China).

### Cell proliferation assays

A CCK-8 assay (Dojindo Laboratories, Kumamoto, Japan) was used to assess cell proliferation. In brief, PTC cells were seeded into 96-well plates at a density of 5000 cells per well. A miR-139-5p inhibition construct was transfected into cells for 24 h. Reagent (10 μL) was added to each well at 0, 24, 48, or 72 h. All plates were assessed by an enzyme-labeling instrument (Thermo Fisher Scientific). Cell proliferation was assessed by the absorbance at 450 nm.

### Cell migration and invasion assays

The migration and invasion abilities of PTC cells were assessed using transwell plates (Millipore, Billerica, MA, USA). PTC were seeded in uncoated (migration assays) or Matrigel-coated (invasion assays) with a diameter of 8 μm (BD Bioscience, Bedford, MA, USA). The upper chamber was seeded with cells at a density of 2 × 10^4^ cells/well in medium without serum, and FBS with 10% serum was added to the lower chamber. For invasion assays, Matrigel-coated chambers were used. After 24 h of incubation, non-migrating cells on the top surface of the filter were removed by rubbing with a cotton swab and cells that had migrated to the lower chamber were quantified in five random fields using an optical inverted microscope at a magnification of 200× (Nikon, Tokyo, Japan).

### RNA immunoprecipitation

According to the manufacturer’s protocol, RNA immunoprecipitation (RIP) was performed in PTC cells 48 h after transfection with the miR-139-5p overexpression construct or miR-NC using the Magna RIP^TM^ RNA Binding Protein Immunoprecipitation Kit (Millipore). Cells (1 × 10^7^) were lysed in RNA lysis buffer, then the cell lysate was conjugated to magnetic beads conjugated to human anti-Argonaute 2 (AGO2) antibody (Millipore) or control mouse IgG (Millipore) in RIP immunoprecipitation buffer. The samples were incubated with proteinase K (Gibco, Grand Island, NY, USA) and IP RNA was isolated. The extracted RNA was examined by reverse transcription PCR to investigate the enrichment of circBACH2.

### Tumor xenograft model and tumorigenicity assay

BALB/c nude mice (*n* = 5 per group) were acquired from Beijing Vital River Animal Company. TCP-1 cells stably expressing circBACH2 or miR-139-5p were suspended (1 × 10^6^ cells/mL) in 100 μL of PBS and subcutaneously injected into the flanks of nude mice. Vernier calipers were used to determine the width and length of tumors each week and the volume was calculated according to the equation: tumor volume = (length × width^2^)/2. Five weeks later, mice were euthanized, and tumors were excised. All animal experiments were conducted in accordance with the Institutional Guidelines for the Care and Use of Laboratory Animals of Pudong New Area Gongli Hospital, Shanghai Second Military Medical University.

### TUNEL assay

For the quantification of apoptosis, TCP1 cells in xenografts were analyzed in situ by a TUNEL assay using the Apoptosis Detection Kit (POD, Roche, Switzerland) according to the manufacturer’s protocol. Slices of the xenografts (3-μm thick) were deparaffinized and rehydrated with xylene and ethanol and permeabilized with 20 μg/mL proteinase K (Gibco), followed by inactivation of endogenous peroxidase with 3% H_2_O_2_. The sections were washed with PBS, then immersed in TdT buffer for 60 min at 37 °C, and incubated with anti-digoxigenin peroxidase conjugate for 30 min, followed by peroxidase substrate. Lastly, slices were counterstained with 0.5% (wt /vol) methyl green.

### Statistical analysis

All data are reported as the mean ± SD. The Student’s two-tailed unpaired *t*-test was used to determine differences between two groups. A *p* value <0.05 was regarded as statistically significant.
